# Supplementary material for: TssA–TssM–TagA interaction modulates type VI secretion system sheath-tube assembly in Vibrio cholerae
Source: Nat Commun. 2020 Oct 8;11:5065. doi: 10.1038/s41467-020-18807-9 (PMC7545191; doi:10.1038/s41467-020-18807-9)
Supplement: Supplementary file 1 — Supplementary Information [file 41467_2020_18807_MOESM1_ESM.pdf]

Supplementary Information

TssA-TssM-TagA interaction modulates the type VI secretion system sheath-tube assembly in *Vibrio cholerae*

Stietz et al.

Supplementary Figures 1-5
Supplementary Tables

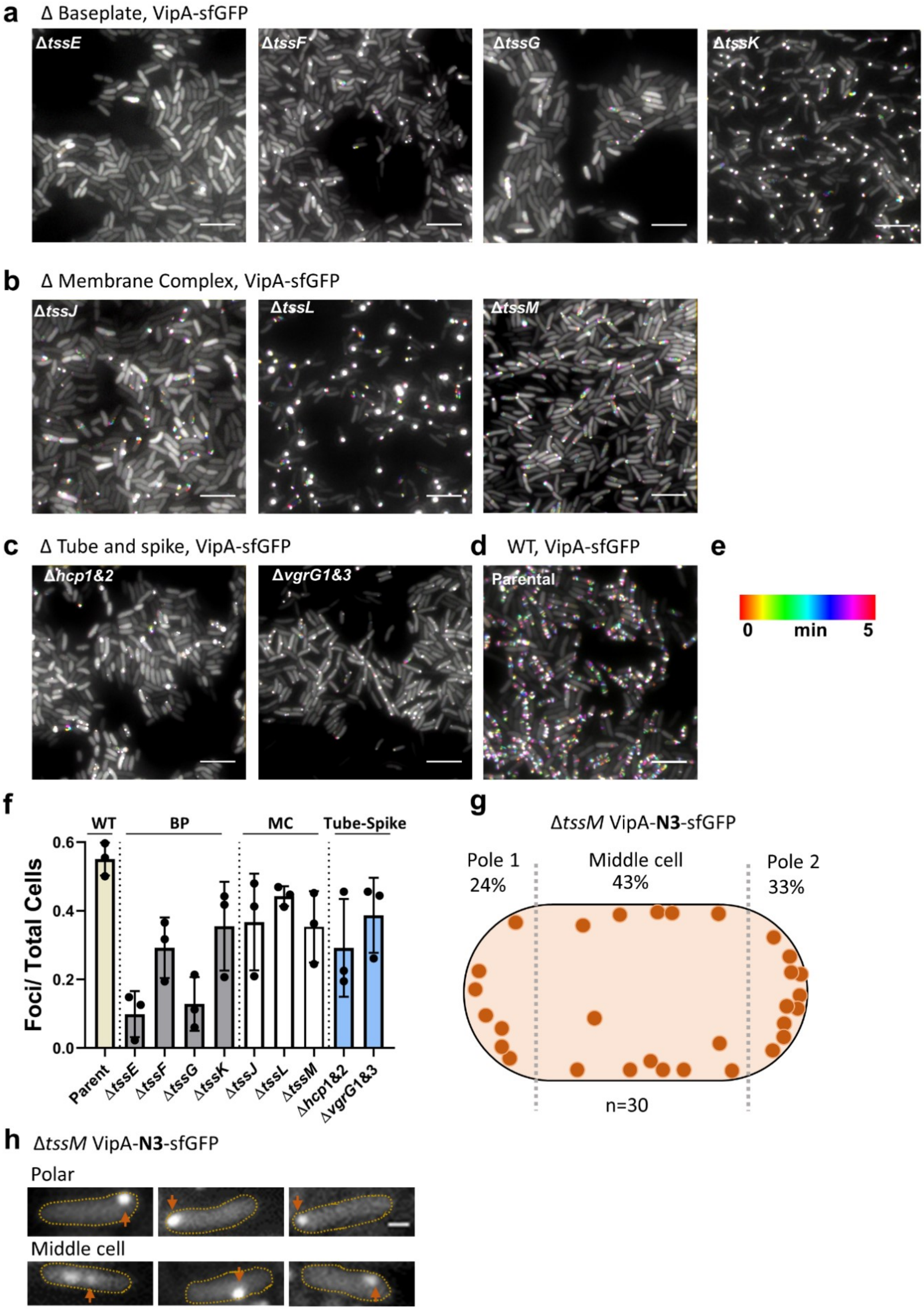
a Δ Baseplate, VipA-sfGFP
ΔtssE
ΔtssF
ΔtssG
ΔtssK
b Δ Membrane Complex, VipA-sfGFP
ΔtssJ
ΔtssL
ΔtssM
c Δ Tube and spike, VipA-sfGFP
Δhcp1&2
ΔvgrG1&3
d WT, VipA-sfGFP
Parental
e
0 min 5
f
WT
BP
MC
Tube-Spike
Foci/ Total Cells
0.0
0.2
0.4
0.6
Parent
ΔtssE
ΔtssF
ΔtssG
ΔtssK
ΔtssJ
ΔtssL
ΔtssM
Δhcp1&2
ΔvgrG1&3
g
ΔtssM VipA-N3-sfGFP
Pole 1
24%
Middle cell
43%
Pole 2
33%
n=30
h ΔtssM VipA-N3-sfGFP
Polar
Middle cell

Supplementary Fig. 1. Deletion of MC or BP impairs sheath assembly in contractile strains. Temporal color-coded images (30 x 30 μm) of contractile VipA-sfGFP strains with baseplate component deletions in **a.** membrane complex deletions in **b.** and Hcp tube and VgrG spike deletions in **c. d.** Temporal color-coded image of contractile VipA-sfGFP parental strain. All scale bars, 5 μm. **e.** Color-coded (spectrum) time scale corresponds to images in a-d. **f.** Number of foci over total cells is indicated for each deletion strain in the contractile VipA-sfGFP background (wild type, WT, yellow; BP, baseplate, gray bars; MC, membrane complex, white bars; Tube-Spike, light blue bars). Each data point represents an independent biological replicate (*n*= 3). Error bars show the mean value ± SD of 3 biological replicates. **g.** Schematic indicating the initiation point of 30 non-contractile VipA-N3-sfGFP sheaths (orange dots) formed in the Δ*tssM* deletion strain in 3 independent experiments. Dashed lines demarcate cell pole areas. The percentage of sheath initiations observed in each cell area is indicated at the top. **h.** Priming region of non-contractile sheaths polymerizing in the Δ*tssM* deletion strain. Cropped cells correspond to VipA-N3-sfGFP signal in grayscale at time 0 of sheath polymerization. Cells are representative of 3 independent experiments. Yellow dashed lines show cell outlines, orange arrows indicate sheath initiation points. Scale bar 0.5 μm. Examples of sheath polymerization in non-contractile Δ*tssM* are included as Supplementary Movie 1.

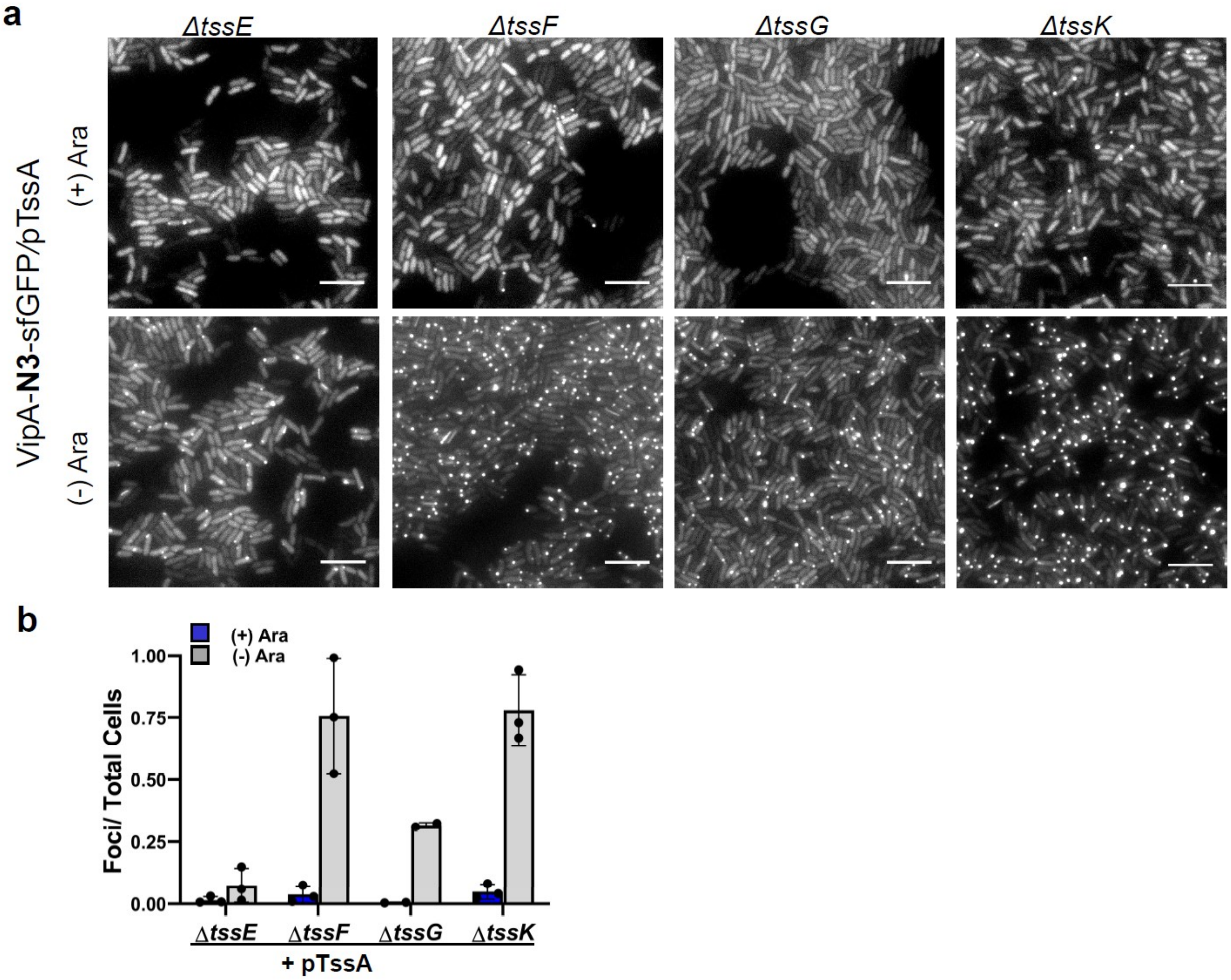

Supplementary Fig. 2. Overexpression of TssA reduces foci formation in BP mutants. a. Still images (30 x 30 µm) of deletion strains of the baseplate components TssE, TssF, TssG and TssK in the non-contractile background VipA-N3-sfGFP with (top) or without (bottom) induction of pTssA plasmid with 0.4% L-arabinose. Scale bars, 5 µm. **b.** Number of foci over total cells counted for each baseplate deletion strain carrying the pTssA plasmid in the presence (blue bars) or absence (gray bars) of L-arabinose. Each data point represents an independent biological replicate (*n*=3). Error bars show the mean value ± SD of 3 biological replicates.

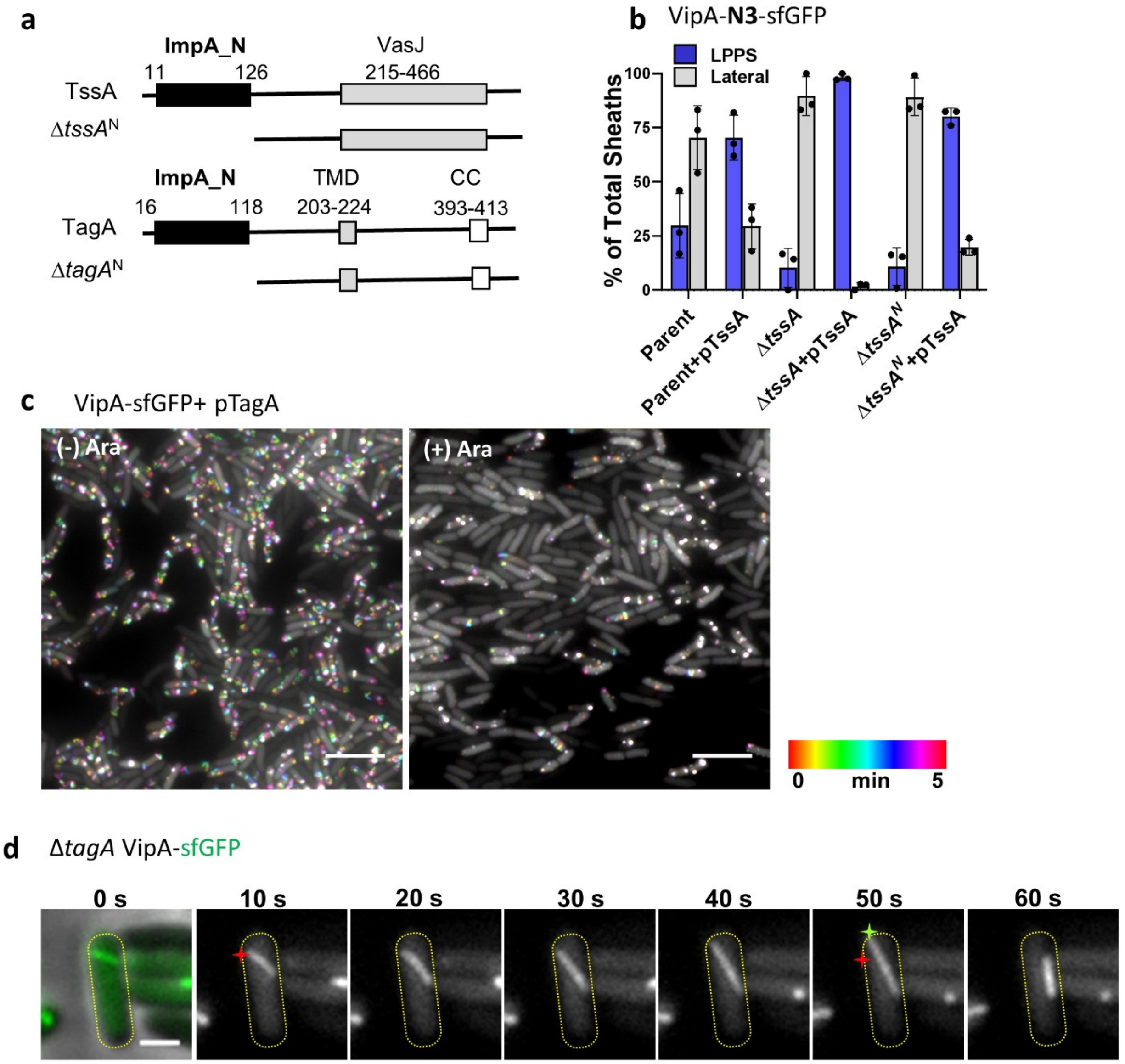
a
ImpA_N
VasJ
11
126
215-466
TssA
ΔtssA^N
ImpA_N
TMD
CC
16
118
203-224
393-413
TagA
ΔtagA^N
b
VipA-N3-sfGFP
LPPS
Lateral
% of Total Sheaths
100
75
50
25
0
Parent
Parent+pTssA
ΔtssA
ΔtssA+pTssA
ΔtssA^N
ΔtssA^N +pTssA
c
VipA-sfGFP+ pTagA
(-) Ara
(+) Ara
0
min
5
d
ΔtagA VipA-sfGFP
0 s
10 s
20 s
30 s
40 s
50 s
60 s

Supplementary Fig. 3. TagA overexpression inhibits sheath formation. a. Diagram illustrates the ImpA_N domain deletion strains used for assays depicted in Fig. 4. **b.** Percentage of total sheaths assembled from cell pole to cell pole (LPPS, blue bars) versus across the cell diameter (lateral, gray bars) in the non-contractile VipA-N3-sfGFP strains quantified before and after induction of pTssA plasmid. Each data point represents an independent experiment (*n*= 3). Error bars show the mean value ± SD of 3 biological replicates. **c.** Temporal color-coded images of VipA-sfGFP cells before (left) and after (right) induction of pTagA plasmid with 0.1% L-arabinose for 15 min. Images are representative of 3 independent experiments and correspond to a 5 min time lapse video. Scale bar, 5 µm. The color-coded (spectrum) time scale is shown at the bottom right. **d.** Image sequence of the contractile VipA-sfGFP TagA deletion strain (Δ*tagA*). Image is a representative example of 3 independent experiments. Left panel is a merge of phase and GFP channels. Dashed yellow lines indicate cell outlines. Red and green stars denote initial and drifted anchoring point, respectively. Scale bars, 1 µm.

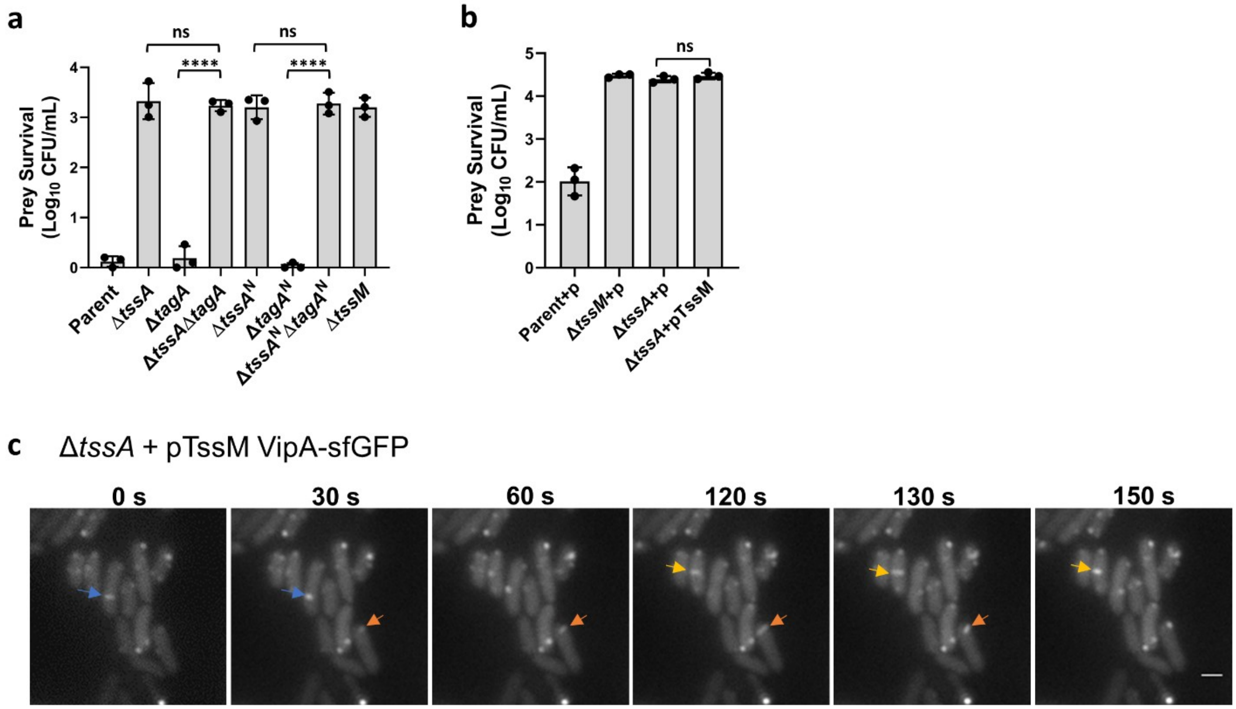

Supplementary Fig. 4. Sheaths assembled in the absence of TssA do not restore T6SS-dependent killing. Relative survival of *E. coli* prey after co-incubation with parental, single and double deletion of *tssA* and *tagA*, and single and double ImpA_N domain deletion killer cells (in **a**) and same prey co-incubated with parental, Δ*tssM* or Δ*tssA* strains carrying empty plasmid (p) or Δ*tssA* complemented with pTssM plasmid (in **b**). Cells were mixed at a 20:1 killer to prey ratio. One-way ANOVA with Sidak's comparing each single deletion to the corresponding double deletion strain (in **a**) or Δ*tssA* strain carrying empty vector versus expressing pTssM (in **b**), ****, $p < 0.0001$, ns, not significant. Each data point represents an independent biological replicate (n=3). Error bars show the mean value ± SD of 3 biological replicates. **c.** Image sequence shows the extension and contraction of three VipA-sfGFP sheaths formed in the Δ*tssA* deletion strain complemented with pTssM. The progression of each sheath is indicated with a different colored arrow (blue, orange, and yellow). Images correspond to GFP fluorescent channel in gray scale. Scale bar, 1 μm.

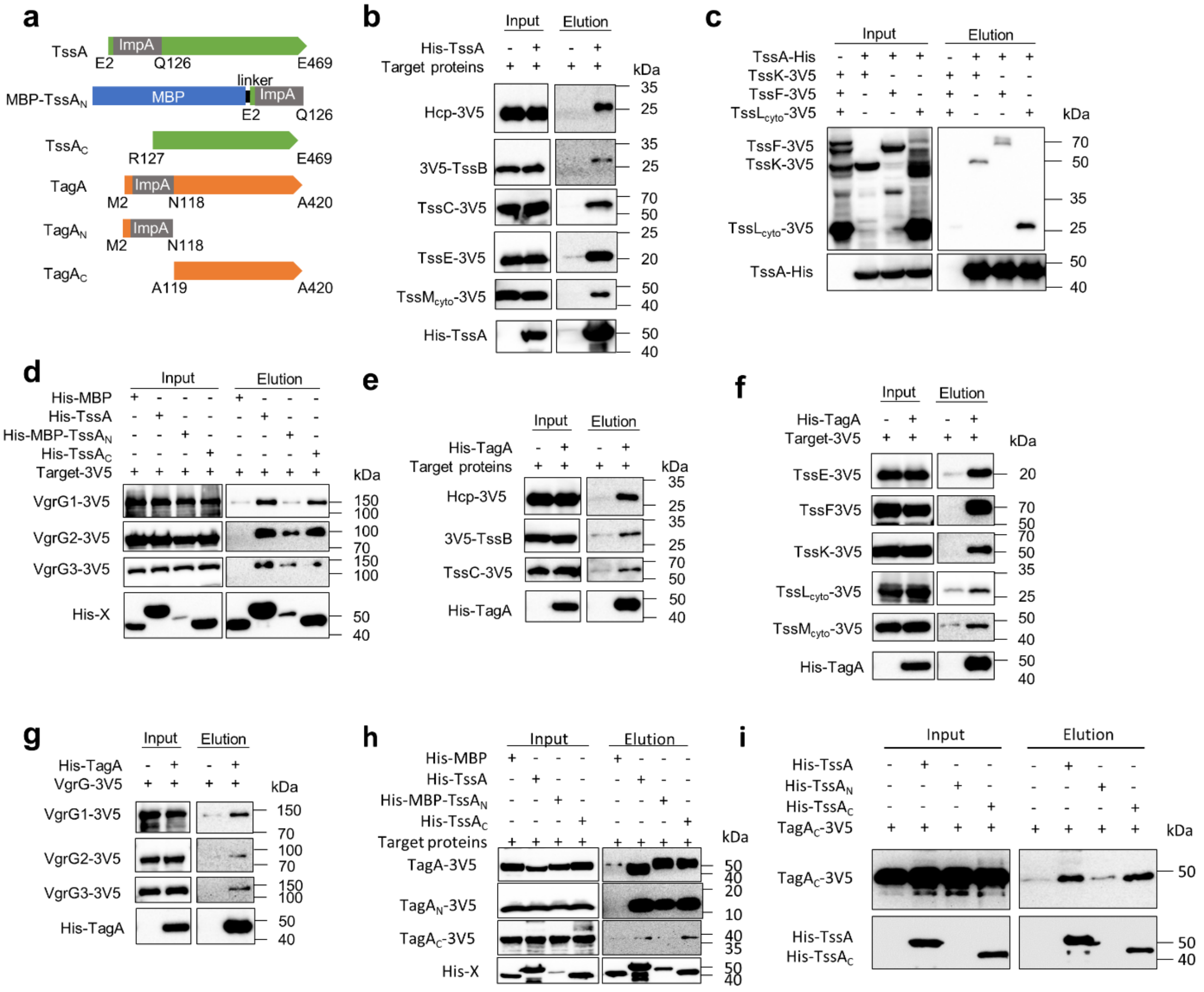

Supplementary Fig. 5. TssA and TagA interact with multiple T6SS structural components. **a.** Schematic showing TssA and TagA constructs used for all pull-down assays in this study. **b, c.** TssA interaction with Hcp tube protein, sheath components TssB and TssC, BP components TssEFK and cytosolic domain of MC components TssM and TssL. **d.** Interaction of TssA full length, N- and C-terminus with the spike proteins VgrG1, 2 and 3. **e.** Interaction of TagA with Hcp tube protein, sheath components TssB and TssC. **f.** Interaction of TagA with BP components TssEFK and cytosolic domain of MC components TssM and L. **g.** Interaction of TagA with VgrG1, 2 and 3. **h, i.** Interactions of TssA with TagA. Note that the TssA N-terminus construct His-tagged maltose binding protein (MBP)-TssA$_N$ but not His-TssA$_N$ showed expression. All experiments were repeated at least 3 times with similar results. Experimental details can be found in Methods section. Uncropped blots are included in the Source Data file.

Supplementary Table 1. Strains

Strain	Genotype	Description	Source
V. cholerae V52	Parental	Serotype 37 clinical isolated from Sudan	[1]
VipA-sfGFP			[2]
VipA-N3-sfGFP			[3]
Δ*tssM* (*vasK)*	VCA0120		[1]
Δ*tssA*	VCA0119		[4]
Δ*tssA*^{N}	VCA0119 [Δ11-126]		This study
Δ*tagA*	VCA0121		[4]
Δ*tagA*^{N}	VCA0121 [Δ16-118]		This study
Δt*ssA;*Δ*tagA*	VCA0119 and VCA0121		This study
Δ*hcp*1&2	VC1415 and VCA0017		[4]
Δ*tssE*	VCA0109		[4]
Δ*tssF*	VCA0110		[4]
Δ*tssG*	VCA0111		[4]
Δ*tssJ*	VCA0113		[4]
Δ*tssK*	VCA0114		[4]
Δ*tssL*	VCA0115		[4]
Δ*vgrG*1&3	VC1416 and VCA0123		[4]
E. coli DH5α λ pir	F- *endA1, glnV44, thi-1, recA1, relA1, gyrA96 deoR, nupG, Φ80dlacZΔM15, (lacZYA-argF)U169, hsdR17(rKmK+), λ-*	Strain used for cloning	New England Biolabs
E. coli SM10 λ pir	Km^{r} *thi-1, thr, leu, tonA, lacY, supE, recA::RP4-2- Tc::Mu, pir*	Strain used for conjugation	Lab stock
E. coli MG1655	Gen^{r}	Prey used for T6SS killing	Lab stock
E. coli BL21 DE3	F$^{–}$ *ompT gal dcm lon hsdS*$_{B}$($r_B^{–}m_B^{–}$) λ(DE3 [*lacI lacUV5-T7p07 ind1 sam7 nin5*]) [*malB*$^{+}$]$_{K-12}$(λ^{S})	Strain used for protein expression	Lab stock
E. coli T-Fast	F- *proA+B+ lacIq* Δ*lacZM15 / fhuA2* Δ*(lac-proAB) glnV galK16 galE15 R(zgb-210::Tn10)TetS endA1 thi-1* Δ*(hsdS-mcrB)5*	Strain used for protein expression	Lab stock

Supplementary Table 2. Plasmids

**Plasmids**	**Description**	**Source**
pDS132, Cm^r	Backbone suicidal plasmid to create in-frame deletions in *V. cholerae*	Lab stock
pDS132-vipA-sfGFP	Suicidal vector to create chromosomal insertion VipA-sfGFP	[3]
pDS132-vipA-N3-sfGFP	Suicidal vector to create chromosomal VipA-N3-sfGFP	[3]
pDS132-Δ*tssA*	Suicidal vector to create chromosomal deletion of VCA0119	This study
pDS132-Δ*tssA*^{N}	Suicidal vector for chromosomal ImpA domain deletion (11-126) of TssA	This study
pDS132-Δ*tagA*	Suicidal vector to create chromosomal deletion of VCA0120	[3]
pDS132-Δ*tagA*^{N}	Suicidal vector for chromosomal ImpA domain deletion (16-118) of TagA	This study
pBAD18, Kan^r	Arabinose-inducible expression backbone vector, kanamycin resistance	Lab stock
pBAD18, Cm^r	Arabinose-inducible expression vector, chloramphenicol resistance	Lab stock
pBAD24, Cm^r	Arabinose-inducible expression vector, chloramphenicol resistance	Lab stock
pBAD24, Kan^r	Arabinose-inducible expression vector, kanamycin resistance	Lab stock
pBAD18-*tssA*	Arabinose inducible expression of VCA0119	This study
pBAD18-*tssM*	Arabinose inducible expression of VCA0120	This study
pBAD18-*tagA*	Arabinose inducible expression of VCA0121	[3]
pBAD33, Cm^r	Arabinose-inducible expression backbone vector, conferring chloramphenicol resistance	Lab stock
pBAD33-*vgrG1*	Arabinose inducible expression of VC1416 missing toxic domain	This study
pBAD33-*vgrG3*	Arabinose inducible expression of VCA0123 missing toxic domain	This study
pBAD33-PAAR2	Arabinose inducible expression of VCA0284	This study
pETDuet1	IPTG-inducible expression vector, ampicillin resistance	Lab stock
pETDuet2	IPTG-inducible expression vector, ampicillin resistance	Lab stock
pBAD24Cm-His-MBP	For expression of MBP with an N-terminal 6×His tag	This study
pBAD24Cm-His-MBP-$TssA_N$	For expression of MBP-$TssA_N$ with an N-terminal 6×His tag	This study
pETDuet2-His-TssA	For expression of TssA with an N-terminal 6×His tag	This study
pETDuet2-His-$TssA_C$	For expression of $TssA_C$ with an N-terminal 6×His tag	This study
pBAD24Kan-TssA-His	For expression of TssA with a C-terminal 6×His tag	This study
pBAD24Kan-TagA-3V5	For expression of TagA with a C-terminal 3×V5 tag	This study
pBAD24Kan-$TagA_N$-3V5	For expression of $TagA_N$ with a C-terminal 3×V5 tag	This study
pBAD24Kan-$TagA_C$-3V5	For expression of $TagA_C$ with a C-terminal 3×V5 tag	This study
pETDuet1-His-TagA	For expression of TagA with an N-terminal 6×His tag	This study

pBAD24Kan-VgrG1-3V5	For expression of VgrG1 with a C-terminal 3×V5 tag	This study
pBAD24Kan-VgrG2-3V5	For expression of VgrG2 with a C-terminal 3×V5 tag	This study
pBAD18Cm-VgrG3-3V5	For expression of VgrG3 with a C-terminal 3×V5 tag	Lab stock
pBAD24Kan-Hcp-3V5	For expression of Hcp with a C-terminal 3×V5 tag	This study
pBAD18Cm-3V5-TssB	For expression of TssB with an N-terminal 3×V5 tag	This study
pBAD24Kan-TssC-3V5	For expression of TssC with a C-terminal 3×V5 tag	This study
pBAD24Kan-TssE-3V5	For expression of TssE with a C-terminal 3×V5 tag	This study
pBAD24Kan-TssF-3V5	For expression of TssF with a C-terminal 3×V5 tag	This study
pBAD24Kan-TssK-3V5	For expression of TssK with a C-terminal 3×V5 tag	This study
pBAD24Kan-$TssM_{cyto}$-3V5	For expression of TssM cytoplasmic domain with a C-terminal 3×V5 tag	This study
pBAD24Kan-$TssL_{cyto}$-3V5	For expression of TssL cytoplasmic domain with a C-terminal 3×V5 tag	This study

References

1. Pukatzki, S. *et al.* Identification of a conserved bacterial protein secretion system in *Vibrio cholerae* using the *Dictyostelium* host model system. *Proc. Natl. Acad. Sci.* **103**, 1528–1533 (2006).
2. Basler, M. & Mekalanos, J. J. Type 6 Secretion Dynamics Within and Between Bacterial Cells. *Science.* **337**, 815–815 (2012).
3. Stietz, M. S., Liang, X., Wong, M., Hersch, S. & Dong, T. G. Double tubular contractile structure of the type VI secretion system displays striking flexibility and elasticity. *J. Bacteriol.* **202**, e00425-19 (2019).
4. Zheng, J., Ho, B. & Mekalanos, J. J. Genetic analysis of anti-amoebae and anti-bacterial activities of the type VI secretion system in *Vibrio cholerae*. *PLoS One* **6**, e23876 (2011).
