# Supplementary material for: TssA–TssM–TagA interaction modulates type VI secretion system sheath-tube assembly in Vibrio cholerae
Source: Nat Commun. 2020 Oct 8;11:5065. doi: 10.1038/s41467-020-18807-9 (PMC7545191; doi:10.1038/s41467-020-18807-9)
Supplement: Supplementary file 3 — Description of Additional Supplementary Files [file 41467_2020_18807_MOESM3_ESM.pdf]

Description of Additional Supplementary Files

File Name: Supplementary Movie 1

Description: **Initiation and polymerization of non-contractile sheaths in the Δ*tssM* strain.** Video plays VipA-N3-sfGFP non-contractile sheaths polymerizing in the *tssM* deletion strain, 4 examples are shown. Each example corresponds to a 5 min time-lapse acquisition and plays at a frame rate of 10 sec per frame. Scale bar 1 µm. A merge of GFP and brightfield channels is shown.

File Name: Supplementary Movie 2

Description: **Assembly of LPPS in *V. cholerae* contractile Δ*tssM* complemented strain.** Video shows the VipA-sfGFP labeled *tssM* strain (Δ*tssM*, left), and the complemented strain (Δ*tssM/p*TssM, right) corresponding to the still images shown in Fig. 2a. Video was taken during 5 min and plays at a rate of 10 frames per second. GFP fluorescence channel is shown in grayscale. Each Fiel d of View (FOV) is 30 x 30 µm. Scale bar, 5 µm.

File Name: Supplementary Movie 3

Description: **T6SS sheath-tube assembly in *V. cholerae* Δ*tssA* deletion and ImpA_N domain deletion.** Sheath assembly in the VipA-sfGFP *tssA* (Δ*tssA*, left), or ImpA_N domain deletion (Δ*tssA*^{N}, middle) and parental (right) strains of *V. cholerae* are shown. Video corresponds to 5 min time-lapse images acquired at a 10 sec frame rate also shown as temporal color-coded images in Fig. 4d. Vi deo plays at a rate of 10 frames per second. GFP fluorescence channel in grayscale is shown. Each Fiel d of View (FOV) is 30 x 30 µm. Scale bar, 5 µm.

File Name: Supplementary Movie 4

Description: **Contractile sheaths formed in the absence of *tagA* and ImpA_N domain deletion in *V. cholerae*.** VipA-sfGFP labeled *tagA* deletion (Δ*tagA*, left), or ImpA_N domain deletion (Δ$tagA^N$, middle) and parental (right) strains of *V. cholerae* are shown. Images correspond to 5 min time-lapse videos acquired at a frame rate of 10 sec per frame. Related to Fig. 4g. Video plays at a rate of 10 frames per second. GFP fluorescence channel in grayscale is shown. Each Field of View (FOV) i s 30 x 30 μm. Scale bar, 5 μm.

File Name: Supplementary Movie 5

Description: **Contractile T6SS sheath-tube assembly in the double Δ*tssA tagA* deletion mutant in *V. cholerae*.** VipA-sfGFP labeled *tssA* deletion only (Δ*tssA*, left) or double deletion (Δ*tssA*Δ*tagA*, right). Videos correspond to temporal color-coded images on Fig. 5a, 5 min time-lapse acquired at a frame rate of 10 sec per frame. Videos play at a rate of 10 frames per second. GFP fluorescence channel in grayscale is shown. Each Field of View (FOV) is 30 x 30 μm. Scale bar, 5 μm.

File Name: Supplementary Movie 6

Description: **Contractile T6SS sheath-tube assembly in the double Δ*tssA tagA* ImpA_N deletion in *V. cholerae*.** VipA-sfGFP labeled *tssA* ImpA_N deletion only (Δ$tssA^N$, left) or double *tssA tagA* ImpA_N deletion (Δ$tssA^N$ Δ$tagA^N$, right). Videos correspond to temporal color-coded images on Fig. 5b, 5 min time-lapse acquired at a frame rate of 10 sec per frame. Video plays at a rate of 10 frames per second. GFP fluorescence channel in grayscale is shown. Each Field of View (FOV) i s 30 x 30 μm. Scale bar, 5 μm**.**

File Name: Supplementary Movie 7

Description: **Restoration of sheath assembly in the Δ*tssA* deletion strain after complementation with pTssM.** Video shows the assembly of VipA-sfGFP labeled sheaths in the *tssA* deletion strain after complementation with pTssM plasmid (right). No vector control Δ*tssA* strain is shown in the left. Videos correspond to temporal color-coded images in Fig. 5c and was acquired during 5 min at 10 seconds per frame. Video plays at a rate of 10 frames per second. GFP fluorescence channel in grayscale is shown. Each Field of View (FOV) is 30 x 30 μm. Scale bar, 5 μm.
