# Supplementary material for: TssA–TssM–TagA interaction modulates type VI secretion system sheath-tube assembly in Vibrio cholerae
Source: Nat Commun. 2020 Oct 8;11:5065. doi: 10.1038/s41467-020-18807-9 (PMC7545191; doi:10.1038/s41467-020-18807-9)
Supplement: Supplementary file 11 — Reporting Summary [file 41467_2020_18807_MOESM11_ESM.pdf]

nature research

Corresponding author(s): Tao Dong

Last updated by author(s): Aug 20, 2020

Reporting Summary

Nature Research wishes to improve the reproducibility of the work that we publish. This form provides structure for consistency and transparency in reporting. For further information on Nature Research policies, see our Editorial Policies and the Editorial Policy Checklist.

Statistics

For all statistical analyses, confirm that the following items are present in the figure legend, table legend, main text, or Methods section.

n/a	Confirmed	
☐	☒	The exact sample size (n) for each experimental group/condition, given as a discrete number and unit of measurement
☐	☒	A statement on whether measurements were taken from distinct samples or whether the same sample was measured repeatedly
☐	☒	The statistical test(s) used AND whether they are one- or two-sided<br>*Only common tests should be described solely by name; describe more complex techniques in the Methods section.*
☐	☒	A description of all covariates tested
☐	☒	A description of any assumptions or corrections, such as tests of normality and adjustment for multiple comparisons
☐	☒	A full description of the statistical parameters including central tendency (e.g. means) or other basic estimates (e.g. regression coefficient) AND variation (e.g. standard deviation) or associated estimates of uncertainty (e.g. confidence intervals)
☐	☒	For null hypothesis testing, the test statistic (e.g. F, t, r) with confidence intervals, effect sizes, degrees of freedom and P value noted<br>*Give P values as exact values whenever suitable.*
☒	☐	For Bayesian analysis, information on the choice of priors and Markov chain Monte Carlo settings
☒	☐	For hierarchical and complex designs, identification of the appropriate level for tests and full reporting of outcomes
☒	☐	Estimates of effect sizes (e.g. Cohen's d, Pearson's r), indicating how they were calculated

Our web collection on statistics for biologists contains articles on many of the points above.

Software and code

Policy information about availability of computer code

Data collection	NA
Data analysis	Commercial and open source software were used for analysis: GraphPad Prism 8.0.0, Microsoft Office, Microsoft Excel, Fiji (ImageJ), NIS-Elements AR 4.40 (Nikon),

For manuscripts utilizing custom algorithms or software that are central to the research but not yet described in published literature, software must be made available to editors and reviewers. We strongly encourage code deposition in a community repository (e.g. GitHub). See the Nature Research guidelines for submitting code & software for further information.

Data

Policy information about availability of data

All manuscripts must include a data availability statement. This statement should provide the following information, where applicable:

- Accession codes, unique identifiers, or web links for publicly available datasets
- A list of figures that have associated raw data
- A description of any restrictions on data availability

The source data for Fig. 1b, c; 2b, c; 3b, d; 4a-c, f; 5e, g and Supplementary Fig. 1f; 2b; 3b; 4a, b and 5b-i are provided with this paper as Source data files. Any other data supporting the current study are available from the corresponding author on reasonable request.

Field-specific reporting

Please select the one below that is the best fit for your research. If you are not sure, read the appropriate sections before making your selection.

☒ Life sciences ☐ Behavioural & social sciences ☐ Ecological, evolutionary & environmental sciences

For a reference copy of the document with all sections, see nature.com/documents/nr-reporting-summary-flat.pdf

Life sciences study design

All studies must disclose on these points even when the disclosure is negative.

Sample size	No sample size calculation was performed. All experiments were repeated at least 3 times in independent days, as it is standard practice for most microbiology assays. For microscopy images we analyzed the total number of bacteria present in 40x40 um field of views from 3 independent biological replicates which is in accordance with previous publications on T6SS microscopy analysis (Basler et al., 2013).
Data exclusions	No data were excluded from the analysis.
Replication	All results reported in this study were reliably reproduced in at least 3 independent days under the same conditions.
Randomization	This is not a randomized control study, randomization is not conventionally used for in-vitro bacterial studies like this one. All sets of experiments were performed under the same described conditions or protocol.
Blinding	The investigators were not blinded to the sample' identity as it is not applicable to in-vitro bacterial studies. Data sets were treated equally for analysis and the appropriate software was used to avoid any influence of the investigators over the results.

Reporting for specific materials, systems and methods

We require information from authors about some types of materials, experimental systems and methods used in many studies. Here, indicate whether each material, system or method listed is relevant to your study. If you are not sure if a list item applies to your research, read the appropriate section before selecting a response.

Materials & experimental systems

n/a	Involved in the study	
☐	☒	Antibodies
☒	☐	Eukaryotic cell lines
☒	☐	Palaeontology and archaeology
☒	☐	Animals and other organisms
☒	☐	Human research participants
☒	☐	Clinical data
☒	☐	Dual use research of concern

Methods

n/a	Involved in the study	
☒	☐	ChIP-seq
☒	☐	Flow cytometry
☒	☐	MRI-based neuroimaging

Antibodies

Antibodies used	Monoclonal antibodies were purchased from Sigma Aldrich (6His # SAB4600386) and Thermo Scientific (V5 # 37-7500). The secondary antibody (anti-mouse # 7076S IgG HRP linked) was purchased from Cell Signaling Technology (CST) .
Validation	Commercial antibodies have been validated by the manufacturers as described on the websites following the links below:<br>6His: https://www.sigmaaldrich.com/catalog/product/sigma/sab4600386?lang=en&region=CA<br>V5: https://www.thermofisher.com/cn/en/antibody/product/V5-Tag-Antibody-clone-2F11F7-Monoclonal/37-7500
